# Rethinking plastic waste: innovations in enzymatic breakdown of oil‐based polyesters and bioplastics

**DOI:** 10.1002/2211-5463.70120

**Published:** 2025-09-09

**Authors:** Elena Rosini, Nicolò Antonelli, Gianluca Molla

**Affiliations:** ^1^ Department of Biotechnology and Life Sciences University of Insubria Varese Italy

**Keywords:** bioplastics, bioplastics degradation, circular bioeconomy, PET hydrolases, polyethylene terephthalate, protein engineering

## Abstract

The global accumulation of plastic waste, exceeding 360 million tonnes annually, represents a critical environmental challenge due to their widespread use and extreme recalcitrance in natural environments. Furthermore, the end‐of‐life processing of bioplastics, which are often marketed as eco‐friendly, remains problematic, with biodegradation often requiring industrial conditions. Enzyme‐based depolymerization of polyesters, such as polyethylene terephthalate (PET) and bioplastics (e.g., polylactic acid (PLA), poly(butylene adipate‐co‐terephthalate) (PBAT), and polyhydroxyalkanoates (PHAs)), has emerged as a promising alternative, offering a green approach to postconsumer plastic management with a reduced environmental impact and in alignment with circular economy principles. This review summarizes recent advances in enzymatic degradation of oil‐derived and bio‐based polyesters. Key recent developments are discussed including novel high‐throughput screenings, computational workflow for improvement of PET hydrolases and *de novo* design of biocatalysts, microbial platforms, and enzyme‐embedded self‐biodegrading bioplastics. Collectively, these innovations are redefining the role of biocatalysis in tackling synthetic polymer pollution. Looking ahead, the integration of enzymatic depolymerization with upcycling pathways, standardized kinetic metrics, and one‐pot bioprocesses represents a viable strategy for sustainable plastic waste valorization.

## Graphical abstract

Plastic pollution remains a critical environmental challenge, and current mechanical and chemical recycling methods are insufficient to achieve a fully circular economy. This review highlights recent breakthroughs in the enzymatic depolymerization of both oil‐derived polyesters and bioplastics, including high‐throughput protein engineering, *de novo* enzyme design, microbial recycling platforms, and enzyme‐embedded self‐degrading materials.

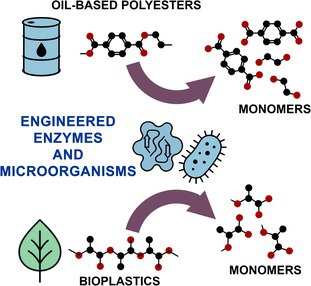

**Abbreviations**
BHET: bis(2‐hydroxyethyl) terephthalate
bio‐PE: bio‐based polyethylene
bio‐PET: bio‐based polyethylene terephthalate
bio‐PP: bio‐based polypropylene
CBM: cellulose‐binding module
CLE1: fungal cutinase‐like enzyme
DepoPETase β: *Is*PETase^D283R/V84L/N233K/F229Y/R280E/F201I/R53Q/D186H^
EG: ethylene glycol
EGAMS: energy‐guided accumulated mutation strategy
FAST‐PETase: evolved PETase
FlashPETase: PHL7^E148K/T158P/S184E/H185Y^
GRAS: generally recognized as safe
HFB4/HFB7: hydrophobins
IC: Intercoil peptide
IM‐proteinase K: immobilized proteinase K
IsPETase: *Ideonella sakaiensis* PET hydrolase
LCC: leaf‐branch compost cutinase
MBP: material‐binding peptide
MHET‐OH: hydroxy‐mono(2‐hydroxyethyl) terephthalate
ML: machine learning
NS: assembled nanospheres
PBAT: poly(butylene adipate‐co‐terephthalate)
PBM: polyhydroxyalkanoate‐binding module
PBS: poly(butylene succinate)
PCL: polycaprolactone
PET: polyethylene terephthalate
PHAs: polyhydroxyalkanoates
PHB: poly(3‐hydroxybutyrate)
PHBV: poly(3‐hydroxybutyrate‐co‐3‐hydroxyvalerate)
PHEs: PET‐hydrolyzing enzymes
PHL7: polyester Hydrolase Leipzig 7
PLA: polylactic acid
PLLA: poly‐l‐lactic acid
SDM: site‐directed mutagenesis
SSM: site‐saturation mutagenesis
SUMO: small ubiquitin‐like modifier
TPA: terephthalic acid
TPA‐OH: fluorescent hydroxy‐TPA

## Addressing the plastic challenge: A sustainable transition from environmental issues to biocatalytic solutions

Plastic pollution represents one of the most pressing environmental challenges of the 21st century. Driven by the massive production and widespread use of synthetic polymers derived from fossil fuels, over 360 million tonnes of plastics are produced annually. A substantial fraction of this plastic accumulates in terrestrial and marine ecosystems, exerting detrimental effects on biodiversity and human health [1, 2]. In response to this environmental issue, the development of bioplastics has obtained significant attention as a sustainable alternative to conventional oil‐based plastics.

**Fig. 1:**
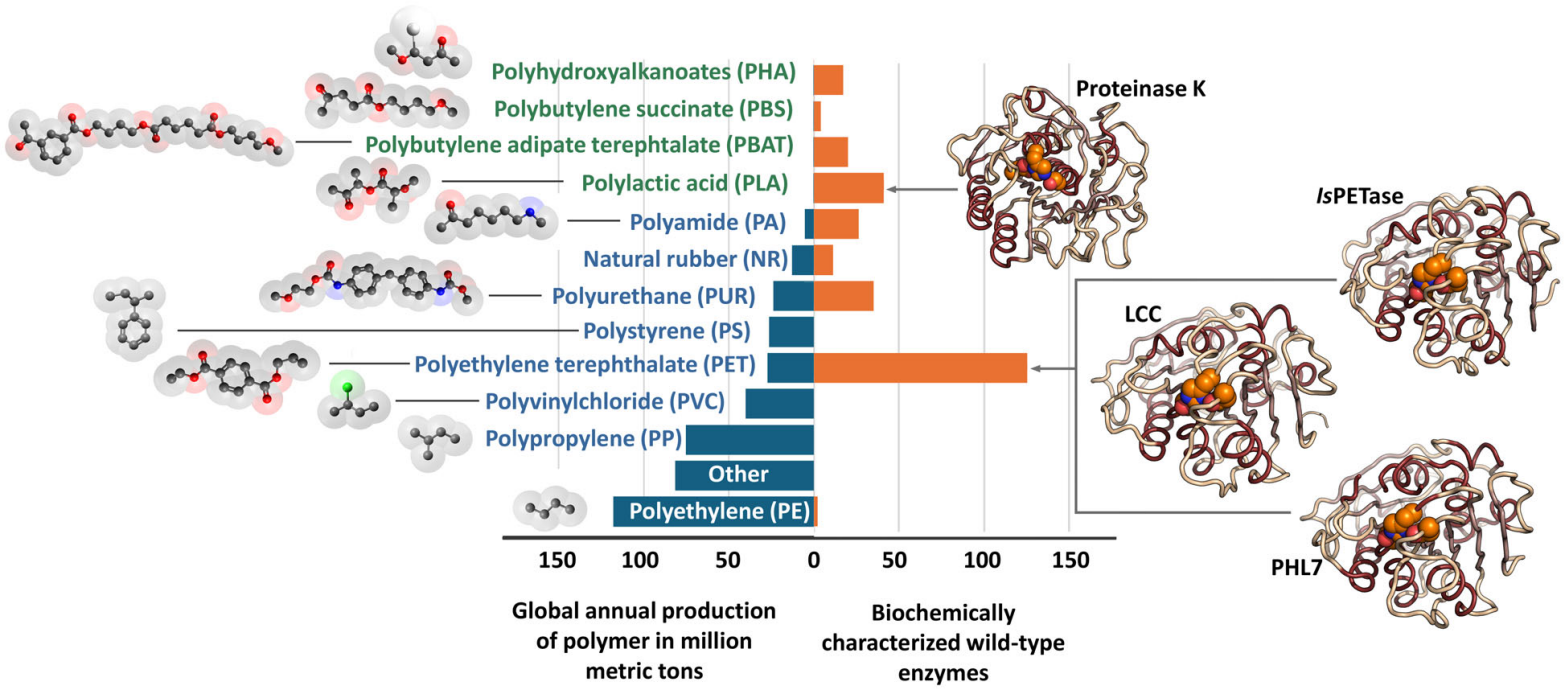
Global production of major plastic polymers and availability of biochemically characterized wild‐type depolymerases. Biodegradable plastics (in green) account for a minor share of global polymer production (overall less than 1 million metric tons per year) but are associated with a relatively higher number of known enzymes. In contrast, conventional fossil‐based polymers (in blue) dominate global plastic production but lack enzymes with confirmed depolymerizing activity. The 3D structures of the following representative enzymes are shown: proteinase K (active on polylactic acid, PLA) (PDB code: 1IC6), and PET‐hydrolyzing enzymes *Ideonella sakaiensis* PET hydrolase, *Is*PETase (PDB code: 6ILW), leaf‐branch compost cutinase, LCC (PDB code: 4EB0), and Polyester Hydrolase Leipzig, PHL7 (PDB code: 7NEI). Data from Pazy database [17].

The term ‘bioplastics’ is conceptually ambiguous, as it encompasses three categories of polymers: (i) bio‐based, nonbiodegradable plastics, such as bio‐based polyethylene (bio‐PE), bio‐based polyethylene terephthalate (bio‐PET), and bio‐based polypropylene (bio‐PP); (ii) fossil‐based biodegradable plastics, such as poly(butylene adipate‐co‐terephthalate) (PBAT) and polycaprolactone (PCL) [3]; and (iii) bio‐based, biodegradable plastics, with a reduced carbon footprint, including polylactic acid (PLA), polyhydroxyalkanoates (PHAs), poly(butylene succinate) (PBS), PBAT, PCL, cellulose, and starch derivatives (Fig. 1). PLA is a bio‐based polyester produced via microbial fermentation of carbohydrate feedstocks, widely employed in rigid and flexible food‐contact materials, PHAs are microbial biodegradable polyesters whose widespread use remains limited by high production costs, and Mater‐Bi, a proprietary family of starch‐based polymers, is extensively used in compostable carrier bags, food packaging, disposable items, and agricultural mulch films [4]. In the present review, the term bioplastics will be used as a synonym for biodegradable plastics. Approximately 43% of bioplastics are biodegradable [3], and about 60% are used in packaging applications. Notably, in 2024, 57.8% of compostable packaging in Italy was effectively collected and treated within the organic waste stream [5]. The adoption of bioplastics is driven by technological advancements and increasingly stringent environmental regulations, particularly in sectors such as packaging, agriculture, and biomedical applications. Currently, bioplastics account for just over 1% of global plastic production; however, the market is expanding rapidly, driven by increasing demand in packaging, automotive, and agricultural sectors, and it is projected to reach USD 98 billion by 2035 [6]. Among European countries, Italy plays a leading role in the production of compostable bioplastics, specifically biodegradable and compostable polymers. In 2022, Italy's national output reached approximately 128,000 tonnes, involving over 271 companies and generating a cumulative turnover of around €1.17 billion [7]. Despite global economic fluctuations, the sector has shown resilience and moderate growth, although a recent slight decrease in this trend has been observed. This is primarily attributable to ongoing discussions regarding the use of reusable fossil‐based plastic products, regulatory ambiguity at the European level, and rising energy prices [8, 9]. Challenges remain concerning cost‐effectiveness, production scalability, and the assurance of complete biodegradability in natural environments [3, 10, 11].

An emerging strategy for mitigating plastic pollution is enzymatic depolymerization. Many bioplastics (e.g., PLA) as well as conventional polyesters like PET share ester linkages in their backbone structures, making them open to enzymatic hydrolysis (Fig. 1). Ester linkages have long been known to be susceptible to attack by various esterases. Indeed, a lipase was one of the first enzymatic activities discovered almost two centuries ago, in 1846, by the French physiologist Claude Bernard [12]. Despite this, the idea of employing enzymes to depolymerize either bio‐ or oil‐based synthetic polyesters remained largely unexplored by the scientific community until the early 2000s. Before that period, only a few studies on this topic were published. For example, in 1981, Williams and colleagues reported on the use of proteinase K from *Tritirachium album* and other enzymes for the enzymatic hydrolysis of PLA [13], while Tanio *et al*. reported on the activity of an extracellular poly(3‐hydroxybutyrate) (PHB) depolymerase from *Alcaligenes faecalis* T1 [14]. Interestingly, they demonstrated for the first time, using solely kinetic analyses, that the active site of the enzyme consisted of four individual subsites, each of them interacting with a different monomer unit of the polymer, a recurring feature of several PET‐hydrolyzing enzymes (PHEs). Exactly twenty years ago, a cutinase from the actinomycete *Thermobifida fusca* was specifically investigated for its ability to depolymerize PET, an oil‐derived synthetic polymer composed exclusively of ester bonds [15]. Eventually, a decade later in 2016, Yoshida *et al*. discovered a novel bacterium, *Ideonella sakaiensis*, isolated from sediment samples from a PET recycling facility in Sakai, Japan [16]. This bacterium was capable of utilizing PET as its primary source of carbon and energy, sparking significant interest in the enzymatic breakdown of polyesters. The main enzyme responsible for this activity, a PET hydrolase (*Is*PETase), is still the most studied PHE to date. Currently, 125 enzymes have been related to PET hydrolysis and 82 to degradation of most bioplastics [17] (Fig. 1).

**Fig. 2:**
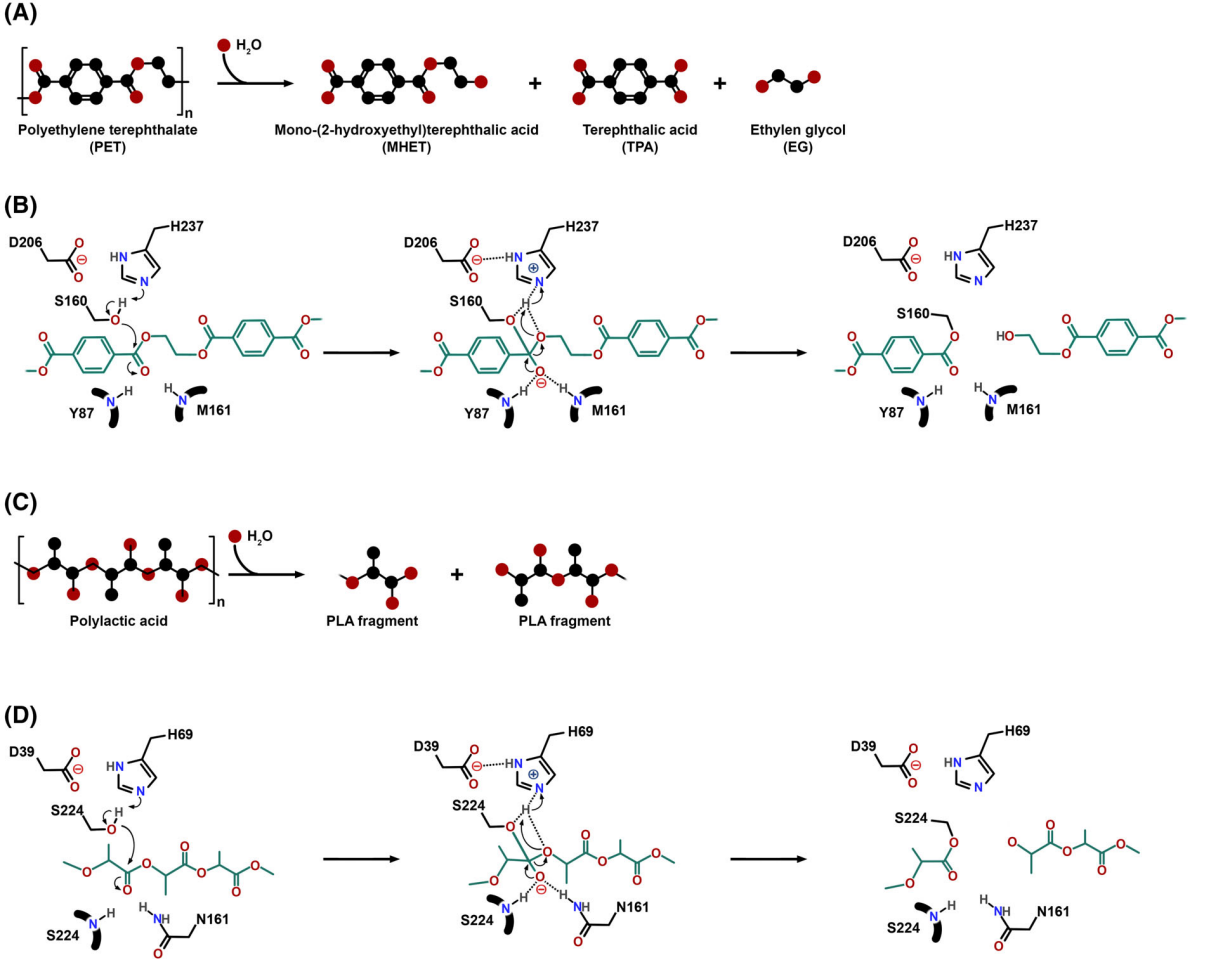
Enzymatic hydrolysis mechanisms of PET and PLA. (A) Polyethylene terephthalate (PET) is depolymerized into mono(2‐hydroxyethyl) terephthalate (MHET), terephthalic acid (TPA), and ethylene glycol (EG). (B) Enzymatic mechanism of PET hydrolysis by *Is*PETase involving the catalytic triad (Ser160, His237, Asp206). The transition state is stabilized by an oxyanion hole formed by Tyr87 and Met161. (C) Polylactic acid (PLA) is enzymatically cleaved into low‐molecular‐weight fragments via ester bond hydrolysis. (D) Enzymatic mechanism of PLA hydrolysis by proteinase K involving the catalytic triad (Ser224, His69, Asp39). The transition state is stabilized by an oxyanion hole formed by Ser224 and Asn161.

All polyester‐degrading enzymes are serine hydrolases belonging to the family of proteinases, lipases, cutinases, and PET hydrolases. Their active site is shaped as a long cleft on the protein surface; the hydrolysis of the ester bond of the polymer is promoted by a canonical serine‐histidine‐aspartate (or glutamate) catalytic triad [18]. In the current review, the reaction mechanism of *Is*PETase is used as a reference model to support the description of the proposed mechanism for the enzymatic PET hydrolysis (Fig. 2A,B). The active site is formed by a region responsible for terephthalic acid (TPA) binding (subsite I) and regions that facilitate the initial binding of the substrate and guide the scissile bond towards the active site (subsite II). During catalysis, the proton is transferred from Ser160 to the leaving group of the substrate via a translational conformation change (the ‘moving histidine’ mechanism) while the Asp206 of the triad forms hydrogen bonds with catalytic His237. During the formation of the transition state, two hydrogen bonds at the oxyanion hole stabilize the PET carboxyl oxygen. The correct substrate binding at the active site is promoted by π‐π interactions between the TPA moiety and the aromatic residues Tyr87 and Trp185, the latter playing a major role in mediating the π‐π interactions because of its flexibility [18].

## Recent trends in PET enzymatic breakdown

PET is a synthetic aromatic polyester widely employed in the production of fibers, textiles, and especially packaging materials. This is due to PET's favorable physicochemical properties such as high tensile strength, chemical resistance, and excellent barrier characteristics. Structurally, PET is composed of repeating units of ethylene glycol (EG) and TPA linked by ester bonds, which confer rigidity and thermal stability to the polymer matrix. The alternating structure of rigid terephthalate rings and flexible EG segments impart PET with a remarkable balance of mechanical strength, thermal stability, and processivity. Overall, enzymatic depolymerization represents a promising way for the sustainable recycling of both conventional plastics like PET and selected bioplastics, complementing existing mechanical and chemical recycling strategies. In this regard, the industrial application of enzyme‐driven processes necessitates robust biocatalysts capable of operating at elevated temperatures, ensuring high reaction rates and substantial bioconversion yields. These requirements are particularly critical for enzymatic PET depolymerization, where a high catalytic activity is essential to prevent polymer recrystallization at temperatures near the PET glass transition range (*T*_*g*_, 69 °C) [19, 20, 21]. These operational issues have driven the development of countless thermostable variants of *Is*PETase, whose native structure shows insufficient activity and thermal stability for industrial‐scale application. Since the pioneering structural and engineering work by Austin *et al*. in 2018, numerous *Is*PETase variants have been produced. These include TS‐Δ*Is*PET [22], ThermoPETase [23], FASTPETase [24], DuraPETase [25], DepoPETase [26], and HotPETase (*T*_m_ = 82.5 °C), enabling activity near the PET *T*_g_ (Table 1) [27]. From 2020, the leaf‐branch compost cutinase (LCC) emerged as a leading biocatalyst owing to its intrinsic higher thermostability, high reaction rate, and TPA production as the main hydrolysis product [20]. Also, LCC has undergone engineering, though to a lesser extent in comparison to *Is*PETase. These enzyme engineering studies have been thoroughly reviewed [28, 29].

**Table 1:** Polyester‐degrading enzymes mentioned in the present review.

Enzyme	Source	Organism	Amino acid substitutions	Expression	Induction condition	pH	Temp (°C)	Time (h)	Substrate	Titer of TPA (or collective titer of products)	Depolymerization yield (%)	References
*Is*PETase	*Ideonella sakaiensis* 201‐F6	*E. coli* C41(DE3)	W159H/S238F	Intracellular	1 mm IPTG	7.2	30	96	PET coupons with an initial crystallinity of 14.8%	410 μm_products_/μg_enzyme_/day	–	[19]
DepoPETase β	*Ideonella sakaiensis* 201‐F6	*E. coli* BL21‐Gold (DE3)	D283R, V84L, N233K, F229Y, R280E, F201I, R53Q, D186H	Intracellular	Autoinduction medium LS‐5052, 48 h, 20 °C	8.6	50	96	50.25 mg·mL^−1^ postconsumer PET	0.25 mm_products_/μg_enzyme_/day	100	[30]
						9	30	48	Body part of commercial PET bottle (PET‐BP, 17.9% crystallinity)	47.6 ± 4.2 μm_products_	>90	
FlashPETase (PHL7)	Plants compost	*E. coli* – BL21 (DE3)	E148K, T158P, S184E, H185Y	Intracellular	ZYM5052 autoinduction medium	8	72	12	Goodfellow film (Gf‐PET)	–	96	[37]
									postconsumer PET plastic packaging box (pc‐PET)	–	97	
FAST‐PETase+MHETase (scaffolding display)	*Ideonella sakaiensis* 201‐F6	*S. cerevisiae* EBY100	S121E, N233K, R224Q, T140D	Extracellular (yeast surface display)	Galactose‐induced, YNB media	8	30	120	PET film	4.95 mm TPA	100	[44]
LCC	Leaf‐branch compost	*E. coli* – BL21 (DE3)	Wild‐type (wt)	Intracellular	ZYM5052 autoinduction medium, 23 h, 21 °C	8	65	72	Goodfellow film (Gf‐PET, ⌀ = 250–500 μm)	91.3 mg_TAeq_/h/mg_enzyme_	>90	[20]
Immobilized LCC^ICCG^ in protein nanosphere	Leaf‐branch compost	*E. coli* Rosetta‐gami B (DE3)	F243I, D238C, S283C, Y127G	Intracellular	1 mm IPTG, 16–20 h, 18 °C	9	70	72	Untreated postconsumer PET	0.30 g_PcPET_/mg_enzyme_/day	98	[47]
LCC variant (whole‐cell strain)	Leaf‐branch compost	*Clostridium thermocellum* DSM1313	Not specified (LCC variant chromosomally integrated)	Extracellular (whole‐cell surface secretion)	Constitutive expression (genomic integration)	7.4	60	240	Pretreated postconsumer PET	5.2 mm TPA	96.7	[46]
LCC^ICCG^‐Cg‐Def YH	–	*In vitro* fusion protein	L9Y, S19H (binding peptide mutations)	Fused synthetic peptide/enzyme	–	–	65	1	PLA/PS mixture (0.105 mg·mL^−1^)	0.105 mg·mL^−1^ lactic acid	100	[54]
*Rs*PETase1	–	*E. coli* – BL21 (DE3)	Synthetic design (34% identity to LCC)	Intracellular	1 mm IPTG, 16 h, 18 °C	8	50	192	Goodfellow film (Gf‐PET)	4 mm TPA + MHET	20–30	[42]
PHL7	Plants compost	*E. coli* – BL21 (DE3)	Wild‐type (wt)	Intracellular	0.1 mm IPTG, 16 h, 18 °C	8	70	16	Goodfellow film (Gf‐PET, ⌀ = 250–500 μm)	694 μm_products_/μg_enzyme_/day	>90	[35]
							60	48	Postconsumer PET	2.6 μm_products_/μg_enzyme_/day	98.6	
PHL7‐Jemez	Plants compost	*E. coli* – BL21 (DE3)	A35V, Q95Y, T112I, Q175E, H185N	Intracellular	1 mm IPTG, 16 h, 20 °C	8	65	48	Amorphous PET film Goodfellow	13 μm_products_/μg_enzyme_/day	96.5	[38]
HSH‐25 peptide	–	–	*De novo* peptide	–	–	8	60	24	Spin‐coated PET thin films	Alteration to the surface results from peptide activity	Qualitative (partial degradation)	[40]
Proteinase K (immobilized in PLA)	*T. album*	–	–	Embedded in PLA backbone	–	8.5	37	96	PLLA solution‐cast films	Weight loss of 78%	78	[49]
Proteinase K (immobilized in PLA)	*T. album*	–	–	Embedded in PLA backbone	–	–	–	106 days	Pro K@SBA‐15‐PLA film	Complete disintegration in home‐compost conditions, 1.57 times faster than control PLA film	100	[50]
ProteinT^FLTIER^	*Thermus sp*. strain Rt41A	*E. coli* – BL21 (DE3)	Multivariant	Embedded in PCL/PLA blend	ZYM‐5052 auto‐inducible medium, 23 h, 21 °C	–	–	20–24 weeks	Blended PLA/PCL films	Complete disintegration in home‐compost conditions	100	[51]
Lipase B (CALB L)	*Candida antarctica*	–	–	Embedded in PLA backbone	–	7.5	37	6	Extruded PCL films containing CALB powder	Weight loss of 100%	100	[52]
*Bs*AprE^S33T/T99Y/E156S^	*Bacillus subtilis*	*B. subtilis* WB800	S33T, T99Y, E156S	Intracellular	27 °C, 27 h	8	30	16	Dissolved PLLA (12.5 mg·mL^−1^)	4.17 μm_products_/μg_enzyme_/day	–	[55]
PLA1 (JW44‐1708)	*Bacillus licheniformis*	–	–	–	–	8	30	18	PlA powder (low MW)	19.4 mg·mL^−1^ lactic acid	50	[56]
FsC (cutinase)	*Fusarium solani*	*K. phaffii*	Wild‐type (wt)	Extracellular	Methanol	8	50	15	PDLLA	8 g·L^−1^ Lactic acid	80	[57]
CLEns (Fungal cutinase‐like enzyme)	*Cryptococcus* sp. S‐2	*S. cerevisiae*	Variant with secretion signal XYNSEC of *T. reesei xylanase* 2	Extracellular	72 h, 26 °C	8	37	240	10 g·L^−1^ PLA films	9.44 g·L^−1^ lactic acid	37	[48]
Thc_Cut1_koST (cutinase)	*Thermobifida cellulosilytica*	*K. phaffii*	Glycosylation site knock‐out variant	Extracellular	Methanol, 1% (v/v)	8	65	96	PBS films	50 mm products	92	[58]

In this section, we will focus on some of the most innovative and conceptually significant recent developments in the field of enzymatic PET hydrolysis, specifically including three major areas of innovation: (i) the adoption of integrated protein engineering strategies that combine computational design, molecular modeling, and high‐throughput screening to enhance enzyme performance under industrially relevant conditions; (ii) the emergence of *de novo* design approaches that enable the construction of artificial PHE with non‐canonical structures, thus overcoming the intrinsic limitations of naturally evolved enzymes; and (iii) the development of microbial platforms allowing increasing biocatalyst stability and reusability.

### Next‐generation biocatalysts for PET depolymerization

An emerging trend in recent studies is the shift from single‐step protein engineering to workflows that integrate multiple computational tools and iterative mutagenesis steps. These strategies exploit diverse complementary online or standalone applications including machine learning (ML) platforms and the combination of different engineering strategies (either rational or directed evolution approaches). This ‘modular’ and ‘stepwise’ organization allows greater simplicity, flexibility, predictive accuracy, and adaptability without demanding specific high‐level computational expertise or hardware.

**Fig. 3:**
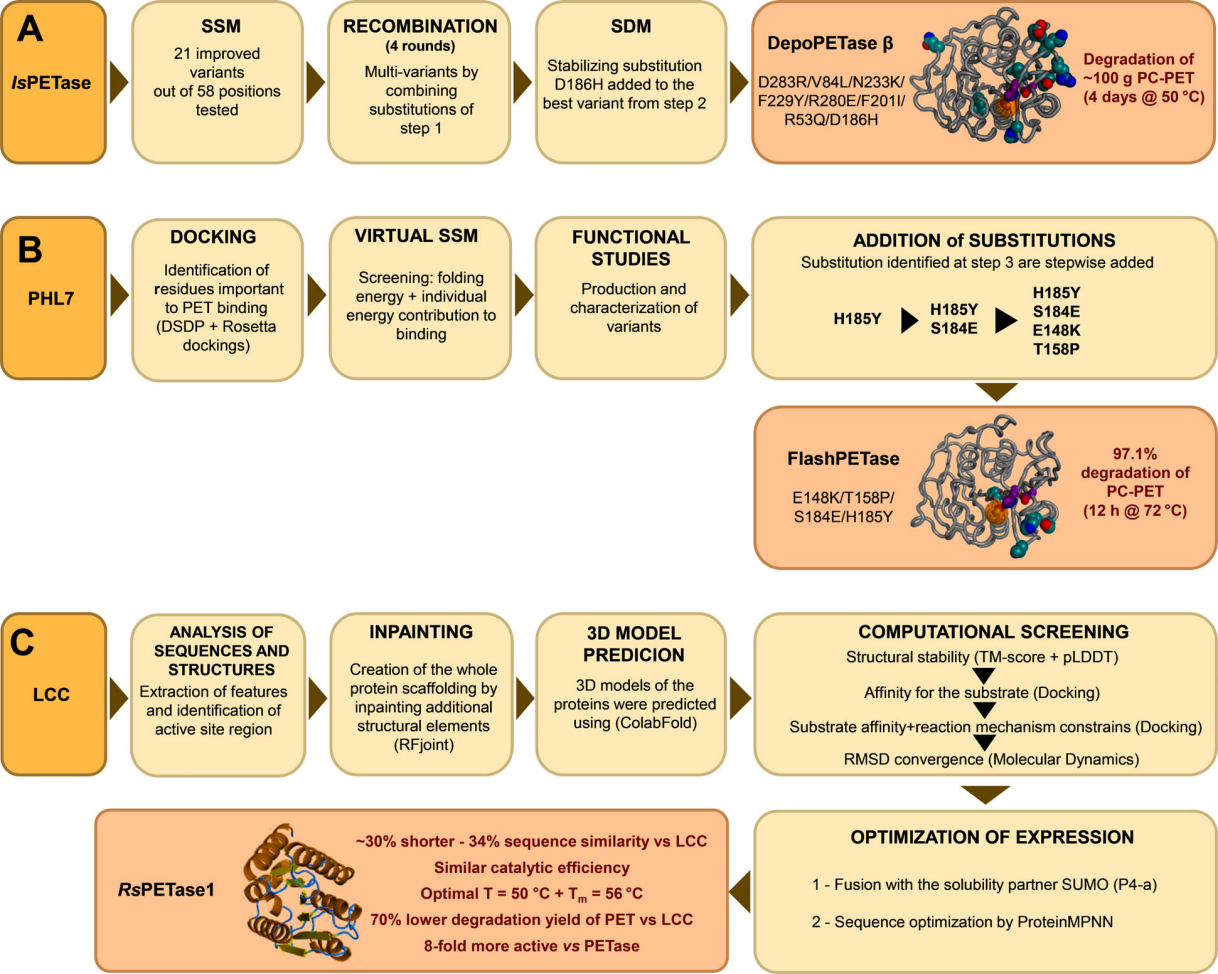
Engineering strategies for enhanced PET‐degrading enzymes. (A) *Is*PETase was improved through stepwise strategies: site‐saturation mutagenesis (SSM), recombination of beneficial mutations, and site‐directed mutagenesis (SDM) leading to the creation of DepoPETase β [30]. (B) PHL7 was enhanced via the Energy‐Guided Accumulated Mutation Strategy (EGAMS) approach including docking, virtual SSM, and functional studies, to produce FlashPETase [37]. (C) Starting from leaf‐branch compost cutinase (LCC), a novel smaller enzyme was produced, *Rs*PETase1 [41].

Recently, Gao and colleagues produced an evolved variant of *Is*PETase targeting residues at the β‐sheet core region of the protein (Fig. 3A) (Table 1) [30]. While the first protein engineering studies targeted active site residues or, to a lesser extent, surface residues of PHEs, only a few approaches targeted this very conserved region of the protein, which represents a challenging task for protein engineering [31]. The authors identified 21 beneficial amino acidic substitutions at 10 different positions in the β‐sheet core of the enzyme taking advantage of a newly developed high‐throughput assay based on the detection of fluorescent hydroxy‐TPA (TPA‐OH) produced from the substrate hydroxy‐mono(2‐hydroxyethyl) terephthalate (MHET‐OH). The amino acid substitutions identified in the first round of mutagenesis were combined through four rounds of iterative recombination and screening. During the process, the screening temperature was gradually increased up to 60 °C, to select for the most stable variants. Eventually, the stabilizing substitution D186H was added to the best variant producing the *Is*PETase^D283R/V84L/N233K/F229Y/R280E/F201I/R53Q/D186H^ (DepoPETase β). This variant exhibited a 22.9 °C increase in thermal stability and showed 12.5‐fold and 179.4‐fold higher activity at 37 °C and 50 °C, respectively, compared to the wild‐type enzyme. DepoPETase β was able to fully degrade 100.5 g of untreated postconsumer PET within 4 days at 50 °C using a 0.26% w/w enzyme loading. During the workflow, the authors took advantage of several bioinformatic tools, namely Consurf (for evolutionary conservation [31]), FoldX (for stability prediction [32]), Schoedinger (for prediction of the structure of variants and molecular docking [33]), and Amber20 (molecular simulations [34]). Bioinformatic analyses showed that the enhanced performances were due to additional noncovalent interactions in the protein β‐sheet core (e.g., hydrogen bonds, salt bridges, tighter residue packing). This resulted in an increased stabilization of the overall protein scaffold, including critical surface loops and the region hosting the catalytic triad.

As an alternative to *Is*PETase and LCC, Polyester Hydrolase Leipzig 7 (PHL7) has recently gained attention due to its properties, which are similar to one of its homologs, LCC [35, 36]. Wang *et al*. (2025) developed a highly efficient PHL7 variant through the novel computational enzyme engineering approach EGAMS (Energy‐Guided Accumulated Mutation Strategy) that integrates molecular docking, molecular dynamics simulations, and binding and folding energy analysis (Fig. 3B) (Table 1) [37]. Substitutions at 18 key residues identified through energy‐based criteria (e.g., folding energy and substrate binding energy) were iteratively introduced and combined to enhance both the catalytic activity and thermostability of the enzyme. After three rounds of *in silico* iterative mutagenesis followed by experimental validation, the authors produced the variant PHL7^E148K/T158P/S184E/H185Y^ (FlashPETase) which exhibited a 2.4‐fold increase in catalytic activity compared to the wild‐type and a melting temperature of 82.9 °C. FlashPETase was able to almost fully depolymerize postconsumer PET films (97.1% of reaction yield) within 12 h at 72 °C without the need for any pretreatment. Interestingly, the crystallinity of the substrate had negligible effect on the performance of FlashPETase: the amount of PET hydrolysis products released from amorphous and high crystallinity (27.3%) PET after 6 h of incubation was similar (52.4 mm and 64.5 mm, respectively). This latter feature further supports the industrial viability of this enzyme. Molecular simulations analyses revealed that the enhanced performance of FlashPETase was linked to multiple factors, including an improved substrate binding, a reduced product inhibition, and an increased structural stability due to a new salt‐bridge network and π‐π interactions at the active site. Notably, the introduction of a proline at position 158 contributed to increase the local structural rigidity, enhancing its thermal stability, while substitutions E148K and S184E provided additional noncovalent stabilizing interactions.

A different multistep approach has been recently applied to the same enzyme by Groseclose *et al*. [38] (Table 1). In the same engineering workflow, the authors combined rational (site‐directed mutagenesis, SDM), semi‐rational (site‐saturation mutagenesis, SSM), and random (DNA shuffling) mutagenesis. In addition, they took advantage of a recently designed high‐throughput screening which coupled a split GFP assay to determine the expression levels of PHL7 variants in the crude extract using bis(2‐hydroxyethyl) terephthalate (BHET) as substrate. This allowed the screening of a very large number of clones without the need for their purification [39]. At first, four improved variants were identified from the first round of DNA shuffling. These variants were submitted to a second round of DNA shuffling together with 11 variants previously obtained by a bioinformatic‐driven SSM approach. The eight best variants from the second round were subjected to a third round of mutagenesis together with the PHL7^Q175E/R205K^ variant which added a stabilizing salt bridge to the protein. This latter variant was obtained by SDM, based on the comparative analysis of the amino acidic sequence of homologous enzymes. Eventually, the best 14 variants of the third round of DNA shuffling were subjected to a last round of mutagenesis producing four improved variants which exhibited significantly enhanced hydrolytic activity on amorphous PET films in comparison to both the native PHL7 and benchmark enzymes such as LCC^ICCG^ (i.e., up to 2.4‐fold higher activity under standard reaction conditions at 70 °C in 1 M potassium phosphate buffer, pH 8.0) [20, 35]. This improvement was also due to the removal of TPA product inhibition. Importantly, variants showed robust activity across a broad pH range (6.0–9.0) and temperature values (65–72 °C, with optimal performance at 70 °C). As an example, in 72 h of reaction at pH 6.0, the PHL7‐Tusas variant reached the same productivity as that obtained at pH 8.0 after 8 h of incubation. Under industrial‐like bioreactor conditions, the PHL7‐Jemez variant showed the highest activity: 2.5‐ to 5‐fold higher initial rates (depending on substrate loading) in comparison to the wild‐type enzyme. Engineered PHL7 variants outperformed LCC^ICCG^ in the depolymerization of amorphous PET film coupons (not PET powder) at 70 °C at their optimal buffer concentration (i.e., 1 M phosphate) while at temperatures below 70 °C and when high crystallinity PET powder was used, LCC^ICCG^ outperformed PHL7 variants. These results highlighted the importance of defining reaction conditions when comparing different PHEs. In addition, the best reaction conditions may not be scalable to industrial levels; for example, 1 M phosphate buffer poses a drawback for the industrial separation of the produced TPA [38].

### Designing next‐generation PET‐hydrolyzing enzymes from scratch

Evolutionary constraints imposed by specific folding architectures significantly limit the diversity of naturally occurring enzymes, thereby reducing the sequence repertoire available for enzyme engineering. Recent breakthroughs in protein structure prediction and *de novo* protein design, powered by ML algorithms, enable the design of enzymes performing the desired functions without relying solely on naturally evolved proteins. Concerning PHEs, the accessible sequence landscape to improve the biocatalyst is limited by the conservation of the canonical α/β structure. To overcome this structural limitation, researchers have *de novo* designed small enzymes adopting an unprecedented, simplified folding thus allowing the development of novel bio‐inspired catalytic systems able to operate under mild conditions. In this context, Koch *et al*. (2024) produced HSH‐25, a *de novo* designed 25‐residue peptide with esterase activity, offering a minimalist and thermostable alternative to traditional PHE [40]. The HSH‐25 peptide is composed of alternating valine and histidine residues, flanking a serine positioned between two histidines (His‐Ser‐His motif) and is folded as a β‐hairpin motif. This motif mimics the canonical serine hydrolase catalytic triads without the structural complexity of naturally evolved PHEs. The predicted unfolding temperature, estimated from molecular dynamics simulations using linear temperature scaling, was 128 °C. Accordingly, circular dichroism spectroscopy revealed no significant conformational changes up to 80 °C, apart from a slight signal variation observed above 75 °C, likely due to partial protein aggregation. Due to the thermophilic behavior, incubation of PET films with HSH‐25 was carried out at 60 °C: atomic force microscopy confirmed the degradation of the PET film surface. Interestingly, this small biocatalyst showed a wide substrate scope with a catalytic activity on different p‐nitrophenyl esters over a broad pH range (7.0–9.5). A different approach to the *de novo* design is exemplified by the work of Ding *et al*. (2025), who reported a robust ML‐driven strategy for engineering novel PHEs (Fig. 3C) (Table 1) [41]. Initially, residues of the active site and its neighbors were isolated from the complete enzyme structure. Subsequently, additional structural elements were inpainted using RF_joint_ (a deep learning method for embedding catalytic motifs into novel protein structures) thus creating the whole protein scaffold. The 3D models of the designed proteins were predicted using ColabFold [42] and the 75 most promising structures were subjected to a computational screening based on MD analysis. Structural stability, affinity for the 2HE‐(MHET)_3_ substrate, reaction mechanism constrains were among the employed criteria used to select the most promising variants. After several rounds of predictions, three novel enzymes have been designed and expressed as small ubiquitin‐like modifier (SUMO) fusion proteins. The best of these synthetic enzymes (P4‐a) showed a hydrolytic activity comparable to the one of LCC. The protein sequence of P4‐a was optimized for the recombinant expression using ProteinMPNN reaching a final protein production of 2.5 mg/L_culture_. The optimized protein, *Rs*PETase1, was ~30% shorter (177 residues) than LCC and shared only 34% sequence similarity with the wild‐type enzyme. It showed a similar catalytic efficiency (*k*_cat_/*K*_m_), an optimal temperature of 50 °C and a lower thermostability (*T*_m_ = 56 °C). Although *Rs*PETase1 showed a 70% lower reaction yield of PET film in comparison with LCC, it was eightfold more active than *Is*PETase. The study not only validates the concept of transplanting functional motifs onto *de novo* protein scaffolds but also underscores the potential of ML in expanding the enzymatic repertoire beyond the limitations imposed by natural evolution. These studies unveiled the potential to circumvent limitations inherent to larger protein scaffolds with peptide‐based catalysts, paving the way for novel biotechnological approaches to plastic recycling.

### Microbial platforms for PET recycling

While the enzyme‐driven PET recycling using free thermophilic enzymes has already reached TRL 8 with the CARBIOS Longlaville plant [43], whole‐cell recycling approaches are still in the early stages of research. In this context, an important goal is the design of biocatalytic platforms able to degrade PET at moderate temperatures, which will be compatible with the use of living cells and of other mesophilic enzymes.

A notable example is provided by Gulati and colleagues, who developed a yeast‐based enzymatic system capable of complete PET depolymerization under mild conditions (30 °C and neutral pH) [44]. A *Saccharomyces cerevisiae* strain was genetically engineered to display on its surface an evolved PETase (FAST‐PETase) [24] and a MHETase for a full depolymerization of PET to TPA and EG. Specifically, a scaffold, consisting of three cohesin domains and a cellulose‐binding domain, was attached to the outer side of the yeast cell wall by disulfide bridges. Recombinant dockerin‐tagged FAST‐PETase and MHETase were then bound to the scaffold through the cohesin domains. Interestingly, the immobilization of two molecules of FAST‐PETase resulted into a 1.8‐fold increase of PET hydrolysis in comparison to the setup composed of only one copy of FAST‐PETase. The further addition of a MHETase led to an additional ~5‐fold boost in product formation. This increase was probably due to the removal of inhibition by the MHET product [45]. When used at 30 °C for 7 days, 4.95 mm TPA was obtained from an amorphous PET film, which is a remarkable result considering the moderate incubation temperature, opening the possibility to efficiently degrade PET under conditions compatible with living cells. Notably, the full activity of the biocatalyst was retained for up to 6 cycles of biodegradation [44].

The reverse approach was employed by Liu *et al*. [46]. Researchers successfully engineered the thermophilic bacterium *Clostridium thermocellum* to stably express improved variants of the thermophilic LCC enzyme, via genomic integration. This approach could circumvent antibiotic supplementation and plasmid instability thus improving continuous expression of the biocatalyst. Different LCC variants have been fused to binding modules to improve their stability and substrate binding; examples of such modules are the cellulose‐binding module (CBM), the polyhydroxyalkanoate‐binding module (PBM), and hydrophobins (HFB4, HFB7). Wild‐type LCC and the HFB7‐LCC variant were integrated and expressed in the host. The microorganism expressing the latter under the efficient P2638s promoter was able to depolymerize ~30% of amorphous PET film in 10 days of incubation at 60 °C. During PET hydrolysis, the pH drops to ~5.6 due to the accumulation of both acidic TPA and acidic metabolites derived from cell growth (e.g., lactate and acetate). Since the activity of LCC is significantly lower at acidic pHs, the reaction was tested in a bioreactor in a controlled pH range. The yield of PET degradation reached ~88.1% and 96.7% after 10 days of incubation at pH 7.0 and 8.0 (an optimal pH range for the biocatalyst and for the microorganism), respectively. The buffering of pH at optimal values for the enzymatic activity and stability demonstrated substantial industrial potential as a cost‐effective and efficient alternative to traditional PET recycling methodologies. Indeed, the products of PET depolymerization can easily be recovered: TPA by precipitation at pH 2.0 and EG through distillation. In addition, since *C. thermocellum* is intrinsically able to degrade cellulose, it will be possible to use this engineered microorganism as a whole‐cell system for the recycling of blended textile waste, formed by cotton (cellulose) and polyester (PET) [46].

López‐Teijeiro *et al*. (2025) developed an innovative *in cellulo* immobilization of LCC^ICCG^.

The enzyme, fused to the *Intercoil* (IC) peptide (LCC^ICCG^‐IC), was incorporated into muNS‐Mi (a truncated form of the avian reovirus scaffold protein) self‐assembled nanospheres (NS) [47]. This system allowed for the direct recovery of functional protein‐loaded particles through mechanical lysis and centrifugation, circumventing chromatographic purification and facilitating applications in biocatalysis, protein engineering, and synthetic biology. NS including LCC^ICCG^ were successfully purified with a yield of LCC^ICCG^ expression ~30% higher in comparison with the free enzyme. The thermal stability was improved, with 40% residual activity after 48 h at 70 °C (*vs*. 13% for the free enzyme) and more than twice the residual activity after 3 months at room temperature in comparison with the free enzyme (58% and 25%, respectively). Notwithstanding a slight decrease (28%) in activity on BHET, the immobilized variant exhibited a wider pH and temperature profile with increased activity at pH <8.0 and temperatures <50 °C in comparison to the free enzyme. Indeed, the performance of NS LCC^ICCG^ at 50 °C was higher with more than 25% of total products being released. Interestingly, NS‐LCC^ICCG^ showed a different product formation ratio between TPA and MHET, with a higher TPA fraction in comparison to the free enzyme. Importantly for potential biotechnological applications, the enzyme showed only a moderate loss of activity after 10 consecutive cycles of PET hydrolysis. Accordingly, enzyme reutilization and replacement of the reaction buffer every 24 h enabled nearly complete depolymerization of low crystallinity postconsumer PET (>90% of weight loss) performed in the 50‐70 °C temperature range. These results support IC fusion as a reliable and versatile strategy for generating immobilized PHEs that retain high catalytic activity and can be efficiently reused, making it well‐suited for sustainable plastic waste bioconversion applications.

## Cutting‐edge technologies for bioplastic breakdown

Despite the optimistic labeling of bioplastics as biodegradable, their actual degradation in soil, freshwater, and marine ecosystems remains inconsistent and inefficient. Factors including temperature, humidity, microbial community composition, and polymer crystallinity heavily influence degradation rates. For instance, PLA degradation requires industrial composting facilities that provide controlled temperature (around 60 °C) and humidity to achieve effective breakdown within months. Similarly, PBAT and PHB may persist longer than expected under natural conditions, which poses challenges for their end‐of‐life management and environmental impact mitigation. Traditional disposal and waste management strategies for these biodegradable plastics include mainly industrial composting (about 40%) [48] and, to a lesser extent, incineration and mechanical recycling. Industrial composting facilities accelerate biodegradation but are limited by the availability of infrastructure and strict operational parameters [49]. Incineration offers energy recovery but generates greenhouse gases and other pollutants, contradicting sustainability goals. Mechanical recycling, though valuable for conventional plastics, often lead to downcycling for biodegradable polymers, resulting in reduced mechanical properties and shorter product lifetimes after each recycling cycle. These limitations have spurred growing interest in alternative approaches such as biological depolymerization and upcycling.

### Built‐in biodegradation: enzyme‐activated self‐degrading plastics

One of the most promising innovations in the design of novel sustainable living polymers is the direct integration of enzymes within bioplastics, named enzyme‐embedded bioplastics. In comparison to conventional biodegradable plastics that often require industrial composting conditions, enzyme‐embedded plastic materials are designed to autonomously activate their end‐life degradation process, even in natural ambient conditions significantly reducing the environmental resident time of the material. Indeed, the *in situ* depolymerization can be activated by humidity, pH variations, or interactions with soil microorganisms.

**Fig. 4:**
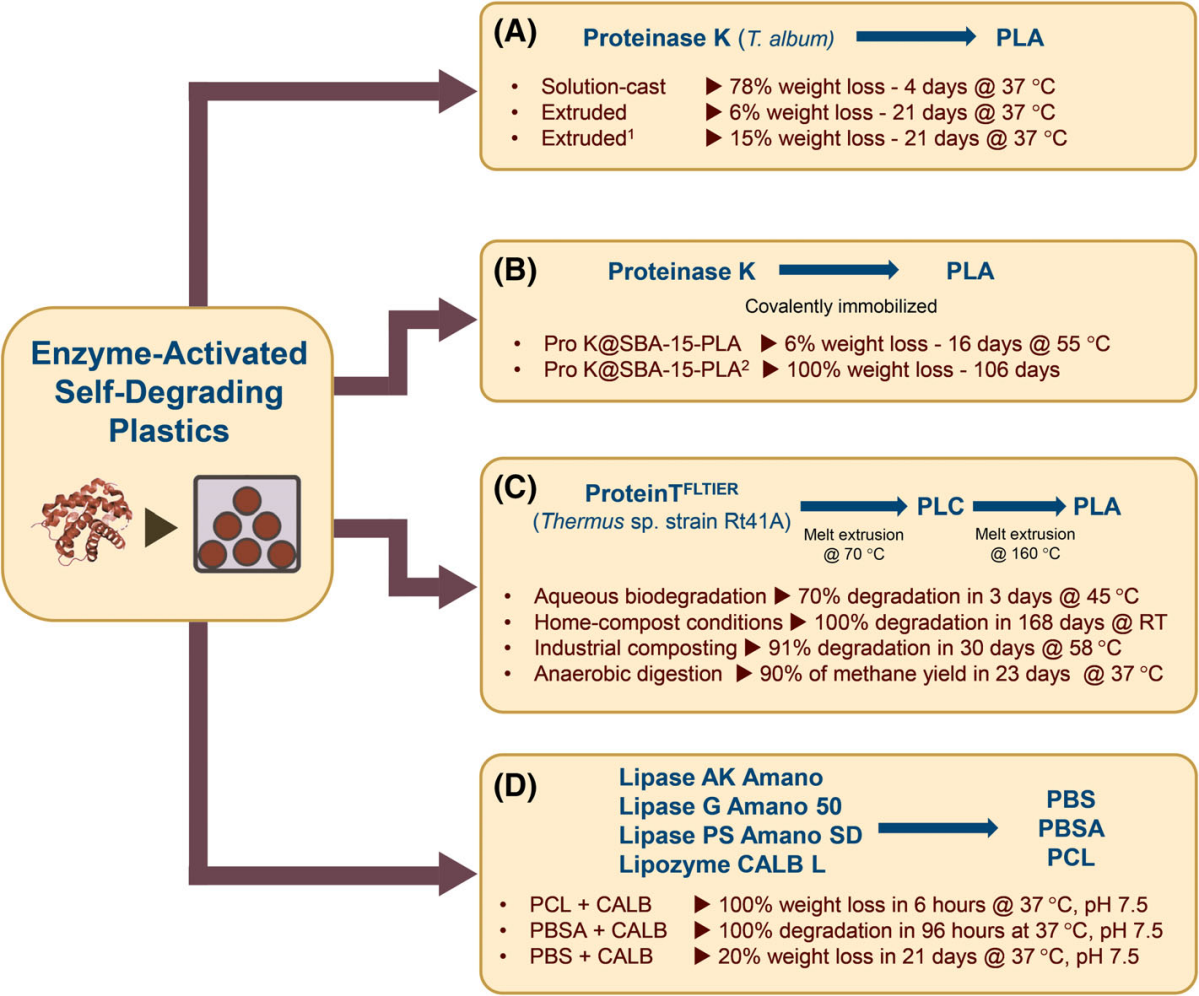
Enzyme‐activated self‐degrading plastic systems. (A) Proteinase K from *T. album* in polylactic acid (PLA) [50]. (B) Covalently immobilized Proteinase K (Pro K@SBA‐15) in PLA [51]. (C) ProteinT^FLTIER^ variant from *Thermus* sp. in PLA [52]. (D) Commercial lipases in polycaprolactone (PCL), poly(butylene succinate‐*co*‐adipate) (PBSA), and poly(butylene succinate) (PBS) [53]. ^1^Poly‐l‐lactic acid (PLLA) extruded films with 1.5% embedded IM‐proteinase K; ^2^Composting conditions.

In this context, Huang *et al*. (2020) incorporated proteinase K in PLA through solution‐cast and melt extrusion techniques without affecting the enzymatic activity. The resulting enzyme‐embedded PLA solution‐cast films displayed a weight loss of 78% after 96 h of incubation at 37 °C (Fig. 4A), while no degradation at all was observed with PLA solution‐cast film without proteinase K [50]. SEM images revealed the formation of pores and holes in the internal structure of the polymer during the first 24 h, suggesting the starting of hydrolysis from the inner core and, subsequently, extending to the surface of the material. The homogeneous distribution of the enzyme inside the polymeric structure, allowed the hydrolysis process to simultaneously start at multiple points of the material, instead of proceeding from the surface, similar to conventional enzymatic treatments of plastics. However, poly‐l‐lactic acid (PLLA) extruded films with embedded proteinase K showed a weight loss of 6% after 21 days. The significant drop in the enzymatic degradation rate between solution‐cast films and extruded films was attributed to the smoother and denser surface of the extruded films, that prevented water to easily penetrate inside and interact with the enzyme, but also to the potential denaturation of the enzyme structure during the melt extrusion process. To overcome these limitations, a previously immobilized proteinase K (IM‐proteinase K) was used to produce extruded PLA films, which showed a 2.5‐fold increase in weight loss in 21 days, suggesting the potential of enzyme immobilization in preventing thermal denaturation and stabilizing enzyme activity. A similar approach was also used by Cao *et al*. (2025), which proposed a strategy to accelerate PLA degradation by embedding immobilized Proteinase K (Fig. 4B) [51]. Unlike the physical integration proposed by Huang, the covalent immobilization allows stable binding of the enzyme to the polymer surface, reducing possible premature enzyme inactivation and improving the process reproducibility. Using the melt extrusion method, the authors obtained a Pro K@SBA‐15‐PLA film, in which the immobilized proteinase K, uniformly dispersed, retained 47% of its initial activity. The complete hydrolysis time in aqueous environments was only 36% lower of the one required under composting conditions, demonstrating the potential of this approach for the industrial degradation of PLA films. In a recent study, a new PLA depolymerase, ProteinT^FLTIER^, a multivariant of an extracellular serine proteinase from *Thermus* sp. strain Rt41A with the best compromise between activity and thermostability, was first incorporated into PCL through melt extrusion at 70 °C, forming an intermediate PCL‐enzyme material that was subsequently integrated into PLA by melt extrusion at 160 °C (Fig. 4C) [52]. The produced enzyme‐PLA film (0.02% w/w enzyme) showed full degradation under home‐composting conditions within 20–24 weeks of incubation. The melt extrusion method was also used to develop self‐biodegradable aliphatic polyesters from PBS, PBSA, and PCL (Fig. 4D). Several commercially available lipases (Lipase AK Amano, Lipase G Amano 50, Lipase PS Amano SD and Lipozyme CALB L), with sufficient thermal stability and high hydrolytic activity for each polyester, were selected and embedded in films of these polymers. All lipases retained their activities after melt extrusion, since even low concentrations (0.07% w/w enzyme) of them embedded in the films led to bioplastic degradation after a few hours [53] (Fig. 4). CALB L embedded extruded films outperformed all the films embedded with the other lipases, suggesting the high potential of Lipozyme CALB L in further improvements of bioplastic degradation via a melt extrusion approach. Notably, CALB powder embedded PCL films achieved complete depolymerization in 3 h, compared to a maximum 50% degradation obtained in 96 h with the other lipases embedded.

The enzymatic hydrolysis of the ester bonds of the polymer backbone is favored by the presence of water in the matrix which can also alter other factors, such as the polymer crystallinity, porosity, and the enzyme mobility within the amorphous phase. The latter is promoted by the absorption of moisture that softens the structure, facilitating the diffusion of the enzyme and the subsequent hydrolysis of the polymer. During the hydrolysis process, one of the most critical aspects is the potential inhibitory effect of the main reaction products, such as lactic acid, which can locally lower the pH and compromise the enzymatic activity. To overcome this limit, some studies proposed the use of buffered enzymes (i.e., enzymes dissolved in a pH‐stabilizing solution to preserve their catalytic activity) or biocatalysts encapsulated in polymeric microgels to maintain a microenvironment suitable to catalysis. Due to their advantages compared to traditional nondegradable bioplastics, such as high degradation efficiency, safety, and versatility, enzyme‐containing materials have been used in different sectors. For example, self‐degrading mulches were successfully tested in agriculture and enzyme‐embedded films were tested in packaging applications where their degradation could be activated only after their use.

In summary, the enzyme‐embedded approach represents a valid and scalable solution to address the problem of environmental bioplastics accumulation, although some challenges still remain. In this scenario, new technologies, like the 3D printing of self‐degradable enzyme‐containing bioplastics, can open new perspectives for tailored biodegradable materials in the environmental and biomedical sectors [54]. Design of economically sustainable systems will require the improvement of the long‐term enzyme stability, release control, and compatibility with industrial conditions.

### Evolving enzymes for faster bioplastic turnover

The approach of bioplastic enzyme degradation is strictly correlated to the improvement of process efficiency and robustness of the biocatalyst by protein engineering. In this context, Lu *et al*. developed an enhanced material‐binding peptide (MBP), Cg‐Def YH (L9Y/S19H), through a directed evolution and rational design approach to minimize experimental work and maximize property improvements [55]. This variant showed a 1.4‐fold higher binding affinity towards PLA than wild‐type and a 2.0‐fold improved specificity on the latter compared to PS. This evolved peptide was fused to LCC^ICCG^ resulting in a 2‐fold enhanced PLA hydrolysis of mixed PLA/PS substrate, compared to LCC^ICCG^, confirming how specific bioplastic hydrolysis can be accelerated through engineered material‐binding peptides [55].

Combining random mutagenesis and computational modeling, Cannon *et al*. generated improved variants of a subtilisin from *Bacillus pumilus* and *Bacillus subtilis* [56]. When tested on PLLA films, these variants showed more than 180‐fold higher catalytic activity compared to the wild‐type enzyme. Structural analyses suggested that the mutations led to a more compact active site and hydrophobic surface, facilitating the penetration of semicrystalline polymers and maximizing the exposure of substrates to the catalytic site. *Bs*AprE^S33T/T99Y/E156S^ showed a 830‐fold increase in activity in comparison with the wild‐type. Interestingly, multiple substitutions synergistically acted to increase depolymerization PLLA. *Bs*AprE^S33T/T99Y/E156S^ produces 71 μm l‐lactate per μg protease, outperforming *Bp*AprE wild‐type.

Recently, Stojanovski *et al*. identified three novel PLA‐degrading enzymes, among more than 100 candidates derived from compost‐isolated strains, by combining functional enrichment and computational screenings [57]. All enzymes showed a promiscuous enzymatic activity also on PBAT, PCL, and other polyesters, making them promising candidates for enzymatic recycling strategies under industrial and environmental conditions. A very interesting aspect with regard to the conservative computational approach was used by the authors. Starting from the genomes of the isolated strains, sequences encoding extracellular enzymes were filtered and used for two strategies: in the first one, the sequences were annotated according to known enzymatic functions to identify functional similarities. In the second one, the proteins were analyzed for the presence of catalytic structural features known to be associated with depolymerase activities. In both cases, the results were cross‐referenced with the characteristics of the enzymes documented in the PlasticDB (a database of plastic‐degrading enzymes). By merging the results, they obtained a smaller set of 105 candidates, thus optimizing the subsequent functional screening phase. By using a high‐throughput screening platform, 97 enzymes were successfully expressed and screened for functional characterization, resulting in 3 active enzymes towards PLA. The three PLA‐degrading enzymes (JW44_1708, JW45_1534, and JW51_1026) were isolated from *Bacillus licheniformis, Caldibacillus hisashii, and Bacillus sanguinis*, respectively. JW44_1708 achieved 50% conversion of solid PLA powder in 18 h at 30 °C under optimized reaction conditions, making it a promising candidate enzyme for ambient temperature enzymatic PLA recycling [57].

These studies demonstrate the feasibility to generate new variants of bioplastic degrading enzymes with improved properties. However, issues related to large‐scale enzymatic production, downstream processing costs, and ecotoxicological safety should be also addressed.

### Engineered microorganisms for bioplastic depolymerization

A promising alternative to traditional strategies based on free purified proteins is the use of engineered microorganisms capable of secreting degrading bioplastic enzymes. Industrial yeasts such as *Komagataella phaffii* (formerly *Pichia pastoris*) and *Saccharomyces cerevisiae* have been used for the heterologous expression of hydrolytic enzymes active on biopolymers such as PLA and PBS. These studies focused on the fermentation and the intrinsic ability of yeasts to secrete high amounts of functional enzymes into the culture medium, thus reducing purification costs and simplifying biotechnological application at an industrial scale.

*Komagataella phaffii* was used by Murguiondo *et al*. (2025) as a host for the recombinant production and secretion of the fungal cutinase FsC, an enzyme known for its efficiency in the degradation of PLA [58]. The secreted enzyme showed high hydrolytic activity towards this polymer, and specifically, towards its amorphous stereospecific form poly(D,L‐lactic acid) (PDLLA). An interesting aspect of the study is that the optimization of secretion and stability in the medium was not reached through the insertion of substitutions in the enzyme itself. The authors obtained hydrolysis yields comparable to or exceeding those obtained with purified enzymes produced in *E. coli* or other bacteria. In 15 h, nearly complete hydrolysis of PDLLA was achieved, producing over 8 g/L of lactic acid from 10 g/L PDLLA at 50 °C.

In 2023, Myburgh and colleagues reported the recombinant expression of a fungal cutinase‐like enzyme (CLE1) in the yeast *S. cerevisiae*, which produced a crude supernatant that efficiently hydrolyzed different types of PLA materials [49]. The Y294[CLEns] strain allowed the hydrolysis of PLA films, with the production of up to 9.44 g/L of lactic acid from 10 g/L of polymer with a mass loss greater than 40% in <2 weeks. This approach has two key advantages: it exploits a well‐known GRAS (Generally Recognized As Safe) microorganism, already widely used in the food and pharmaceutical sectors. In addition, it allows the direct application of fermentation media for enzymatic treatments, avoiding expensive purification or enzymatic immobilization steps. Furthermore, the possibility of modulating growth conditions to maximize secretion made this system adaptable to industrial composting or biorecycling plants. *K. phaffii* was also exploited by Gamerith and collegues as a host for the secretion of the bacterial cutinase Thc_Cut1 from *Thermobifida cellulosilytica*, active towards aliphatic polymers such as PBS [59]. The variant Thc_Cut1_koST and wild‐type Thc_Cut1 achieved 92% and 41% hydrolysis of PBS films, in 96 h at 65 °C, respectively. The secreted enzyme also showed activity on poly(3‐hydroxybutyrate‐co‐3‐hydroxyvalerate) (PHBV), suggesting its potential versatility in contexts of different wastes. Overall, microbial engineering represents a promising strategy for the efficient and safe degradation of bioplastics. The use of engineered strains of well‐known yeast lines offers concrete advantages in terms of both catalytic efficiency and economic sustainability. Thanks to the continuous progresses in genetic engineering and fermentations technologies, these microbial platforms will soon become competitive with current chemical or physical recycling processes.

## Conclusions and future perspectives

The progress in enzyme‐based plastic degradation and upcycling over the past few years has been remarkable, yet significant challenges remain before these innovations can fully realize a circular plastic economy. A key priority for future research and development is the scalable implementation and real‐world deployment of these technologies. Indeed, the enzymatic depolymerization of fossil‐ and bio‐based plastics is transitioning from laboratory innovation to a central pillar of the circular bioeconomy. Recent advances in enzyme discovery, protein engineering, and synthetic biology expanded the feasibility of enzyme‐mediated plastic degradation and upcycling. However, this progress is accompanied by significant challenges that demand interdisciplinary approaches for effective resolution. On the enzymatic front, computational and ML‐guided workflows are revolutionizing the engineering of the required enzymes, leading to variants with higher thermostability, solubility, and catalytic efficiency. The development of *de novo* scaffolds and redesigned active sites has expanded the range of targetable polymers, overcoming evolutionary limitations and enabling enzymatic depolymerization of highly crystalline PET, as well as PLA, PBS, and polyurethanes. Nonetheless, enzyme activity often remains limited to specific substrates and is strongly influenced by environmental factors such as pH and polymer morphology.

Bioplastics, often promoted as inherently degradable, pose their own set of challenges. While materials like PLA and PHAs offer bio‐based alternatives to conventional plastics, their enzymatic hydrolysis efficiency varies widely depending on polymer structure, additives, and environmental context. Recent efforts have expanded the enzymatic toolbox beyond PET, with cutinases, amidases, and oxidative enzymes, derived from insects and extremophiles, showing promising activity on PLA, polyamides, and polyolefins. Furthermore, fungal cocktails, microbial consortia, and insect‐gut engineered systems are being explored for the integrated degradation of mixed plastic waste streams, including thermoplastic starch and biodegradable blends.

Looking forward, several priorities will shape the field: (i) the development of standardized metrics for enzymatic performance to enhance reproducibility [60]; (ii) the reduction of buffer and energy requirements in industrial settings; (iii) harnessing kinetic models and real‐time analytics to optimize reaction pathways; and (iv) discovery of novel enzymes through metagenomics, ancestral reconstruction, and ML‐assisted design [61]. In addition, a growing emphasis is now placed on the integration of depolymerization with upcycling, turning plastic‐derived monomers into high‐value compounds via microbial metabolism or enzymatic cascades [62]. Examples include the synthesis of enantiopure amino acids, biodegradable copolymers, and chemical building blocks. Consolidated one‐pot bioprocesses, where enzyme production, degradation, and conversion occur in the same vessel, are emerging as a scalable strategy to reduce costs, energy inputs, and reliance on harsh chemicals such as NaOH, widely used to buffer pH decrease during polymer hydrolysis.

In conclusion, a new generation of bio‐based recycling technologies capable of transforming plastic waste into a source of renewable resources is emerging. By uniting molecular insight with process innovation, enzymatic degradation of PET and bioplastics is poised to evolve from a promising scientific concept to a viable industrial solution.

## Conflict of interests

The authors declare no competing interests.

## Author contributions

ER and GM conceptualized the review; ER, GM, and NA wrote the final version; NA prepared Table 1. All coauthors have approved the submission of this manuscript.
